# Outcomes of Patients With Cancer With Myocardial Infarction-Associated Cardiogenic Shock Managed With Mechanical Circulatory Support

**DOI:** 10.1016/j.jscai.2023.101208

**Published:** 2023-11-13

**Authors:** Orly Leiva, Richard K. Cheng, Sunil Pauwaa, Jason N. Katz, Jose Alvarez-Cardona, Samuel Bernard, Carlos Alviar, Eric H. Yang

**Affiliations:** a Division of Cardiology, Department of Medicine, New York University Grossman School of Medicine, New York, New York; b Division of Cardiology, University of Washington, Seattle, Washington; c Division of Cardiology, Advocate Christ Medical Center, Oak Lawn, Illinois; d Division of Cardiology, Department of Medicine, Duke University, Durham, North Carolina; e UCLA Cardio-Oncology Program, Division of Cardiology, Department of Medicine, University of California Los Angeles, Los Angeles, California

**Keywords:** acute myocardial infarction, cardiogenic shock, cardio-oncology, mechanical circulatory support

## Abstract

**Background:**

Cardiogenic shock (CS) is the leading cause of death among patients with acute myocardial infarction (AMI) and is managed with temporary mechanical circulatory support (tMCS) in advanced cases. Patients with cancer are at high risk of AMI and CS. However, outcomes of patients with cancer and AMI–CS managed with tMCS have not been rigorously studied.

**Methods:**

Adult patients with AMI–CS managed with tMCS from 2006 to 2018 with and without cancer were identified using the National Inpatient Sample. Propensity score matching (PSM) was performed for variables associated with cancer. Primary outcome was in-hospital death, and secondary outcomes were major bleeding and thrombotic complications.

**Results:**

After PSM, 1287 patients with cancer were matched with 12,870 patients without cancer. There was an increasing temporal trend for prevalence of cancer among patients admitted with AMI–CS managed with tMCS (*P*_trend_ < .001). After PSM, there was no difference in in-hospital death (odds ratio [OR], 1.00; 95% CI, 0.88-1.13) or thrombotic complications (OR, 1.10; 95% CI, 0.91-1.34) between patients with and without cancer. Patients with cancer had a higher risk of major bleeding (OR, 1.29; 95% CI, 1.15-1.46).

**Conclusions:**

Among patients with AMI–CS managed with tMCS, cancer is becoming increasingly frequent and associated with increased risk of major bleeding, although there was no difference in in-hospital death. Further studies are needed to further characterize outcomes, and inclusion of patients with cancer in trials of tMCS is needed.

## Highlights

• Among patients with acute myocardial infarction-cardiogenic shock and temporary mechanical circulatory support, the proportion with cancer has increased.
• Patients with cancer have similar risk of in-hospital mortality as patients without cancer.
• Patients with cancer with acute myocardial infarction-cardiogenic shock and temporary mechanical circulatory support are at higher risk of bleeding.

## Central Illustration

**Figure:**
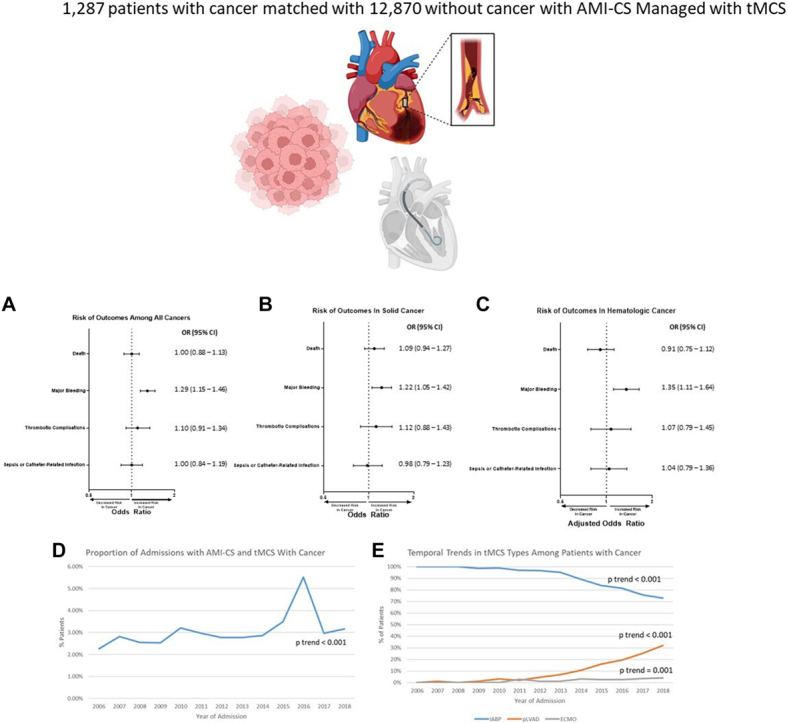

## Introduction

Cardiogenic shock (CS) is a common complication of acute myocardial infarction (AMI), occurring in approximately 4% to 12% of patients, and is associated with significant morbidity and mortality.^1^^,^^2^ Patients with cancer are a growing population at high risk for cardiovascular events due to improvements in cancer-specific outcomes, shared pathophysiology, cancer therapy-associated cardiotoxicity, and accelerated atherosclerosis.^3^ Unsurprisingly, there has been a corresponding increase in the prevalence of AMI and AMI–CS in this cohort.^4^^,^^5^ These patients have worse outcomes and lower rates of invasive interventions such as percutaneous coronary intervention (PCI) and coronary angiography.^6^^,^^7^ There are several potential reasons for reduced use of PCI among patients with cancer including anemia, thrombocytopenia, concern for future bleeding risk of dual antiplatelet, and a concern for poor overall prognosis.^8^

Temporary mechanical circulatory support (tMCS), including intra-aortic balloon pump (IABP), peripheral left ventricular assist device (pLVAD), and extracorporeal membrane oxygenation (ECMO), is often used to support patients with CS as a bridge to recovery, decision, or advanced therapies (durable MCS or heart transplantation).^9^^,^^10^ Patients with cancer and CS pose a clinical conundrum given their paradoxically increased risk of bleeding and thrombosis, both of which are common complications of tMCS. Cardiologists may also be hesitant to manage patients with cancer with invasive procedures given unknown or perceived poor long-term oncologic prognosis, particularly in patients with metastatic disease.^11^ Additionally, patients with AMI–CS are often managed with tMCS as a bridge to recovery, which suggests a possible utility of managing patients with limited long-term survival.^12^ Given the paucity of studies examining outcomes associated with the use of tMCS in cancer patients, we sought to investigate outcomes and trends of these patients admitted with AMI–CS and managed with tMCS using a large, nationwide database.^13^ Additionally, we sought to identify factors associated with tMCS use among patients with cancer and AMI–CS in order to understand nationwide trends in patient selection for tMCS in this patient population.

## Methods

### Study design and population

Hospitalizations for patients with AMI and CS who were managed with tMCS were identified using the National Inpatient Sample (NIS). The NIS is the largest inpatient database in the United States, captures approximately 20% of all inpatient admissions, and is part of the Healthcare Cost and Utilization Project. Data in the NIS are derived from administrative billing data submitted by hospitals to statewide data organizations and contain demographic and clinical characteristics. The NIS used *International Classifications of Diseases, 9th edition* (ICD-9) until September 2015 and subsequently ICD-10. This study was deemed exempt by the New York University Grossman School of Medicine Institutional Review Board given that the NIS is a publicly available and deidentified database.

All hospitalizations with a primary diagnosis of AMI, secondary diagnosis of CS, and any procedure code for tMCS between January 1, 2006 and December 31, 2018 were included. Patients with active cancer were identified using ICD-9 and ICD-10 codes.^14^^,^^15^ Procedures, including IABP, pLVAD, ECMO, left heart catheterization, PCI, vasopressor use, mechanical ventilation, and coronary artery bypass grafting (CABG) were captured using ICD-9 and ICD-10 procedure codes. Comorbidities were captured via ICD-9 and ICD-10 codes and Elixhauser comorbidities.^16^ The ICD-9 and ICD-10 codes used for this study are listed in Supplemental Tables S1 and S2.

### Outcomes

In-hospital outcomes were evaluated for patients with cancer and compared to patients without cancer. Our primary outcome was in-hospital death. Secondary outcomes of interest included major bleeding composite (procedure-related bleeding, intracranial bleeding, gastrointestinal bleeding, or transfusion of blood products), thrombotic complication composite (stroke, arterial thromboembolism, or venous thromboembolism [VTE]^17^), sepsis or catheter-related infections, and their components. Outcomes were abstracted using ICD-9 and ICD-10 codes (Supplemental Table S1).

### Statistical analysis

Admissions with and without cancer were compared, and standardized mean difference (SMD) was calculated for variables before and after PSM. Continuous variables are presented using the mean and SD, and categorical variables are presented as counts and percentages. Imbalances between groups were considered to be significant if the SMD for a given covariable was ≥0.10. A propensity score (the predicted probability of cancer) was calculated using a nonparsimonious multivariable logistic regression. We included age, sex, race, prior myocardial infarction, prior PCI, prior CABG, heart failure, atrial fibrillation, anemia, chronic lung disease, smoking, diabetes, obesity, hypertension, liver disease, peripheral vascular disease, chronic kidney disease (CKD), prior stroke, prior VTE, thrombocytopenia, ST-segment myocardial infarction (STEMI) presentation, cardiac arrest, mechanical complication, tMCS type, PCI, CABG, invasive hemodynamic monitoring, mechanical ventilation, vasopressor use, palliative care consult, do-not-resuscitate (DNR) status, year of admission, and insurance type as covariables. We used a greedy algorithm to propensity score match 1 patient with cancer to 10 patients without cancer using 0.1 SD caliper width. All analyses between patients with cancer and no cancer were performed using a propensity score matched (PSM) cohort. Patients with cancer and no cancer were compared using logistic regression analysis with results presented as the odds ratio (OR) with 95% CI. An analysis of cancer vs no cancer was also performed and stratified by type of cancer (solid and hematologic cancer).

Data in the NIS can be weighted to provide national estimates of the entire United States hospitalized population using the Agency for Healthcare Research and Quality sampling and weighting method. Unweighted counts were used for all statistical analyses with the exception of trend assessments, for which national weighted estimates were used. Temporal trends in number of weighted AMI–CS admissions managed with tMCS with cancer and outcomes were examined using the Mann–Kendall trend test.

To identify risk factors for in-hospital death, major bleeding, and thrombotic complications in patients with cancer who were hospitalized with AMI–CS and managed with tMCS, we compared the characteristics of patients with cancer who had these events with those who did not. Characteristics that differed between groups (*P* < .15) were included in a multivariable logistic regression, with age, sex, and race being covariables in all models.

To identify factors associated with tMCS use among admissions with cancer and AMI–CS, characteristics of those managed with tMCS were compared with those not managed with MCS. Characteristics that differed between groups (*P* < .15) were included in a multivariable logistic regression, with age, sex, and race as covariables.

Analyses were conducted using SPSS version 29.0 (IBM) and Stata version 15 (STATA Corp). A 2-tailed *P* value of <.05 was considered significant.

## Results

### Admission characteristics

A total of 90,708 admissions for AMI–CS were included, of which 42,034 were managed with tMCS. Admissions with a diagnosis of cancer were less likely to be managed with tMCS compared with admissions without (46.8% vs 35.7%, *P* < .001). Among admissions managed with tMCS, the mean age was 65.8 ± 12.2 years, and 13,039 (31.0%) were female. Of the admissions managed with tMCS, 3.1% admissions had cancer. Among unweighted admissions with cancer, 811 (62.2%) had solid cancer, 514 (39.4%) had hematologic malignancies, and 282 (21.6%) and 34 (2.6%) had multiple malignancies. Prior to PSM, admissions with cancer were older (mean age 69.5 vs 65.7 years, SMD = 0.324), less likely to be non-White (28.2% vs 34.3%, SMD = 0.132), and have higher rates of anemia (26.8% vs 18.8%, SMD = 0.192), chronic lung disease (23.8% vs 19.0%, SMD = 0.117), prior VTE (2.8% vs 1.1%, SMD = 0.127), palliative care consult (8.7% vs 5.9%, SMD = 0.106), and DNR status (9.4% vs 6.6%, SMD = 0.106). Patients with cancer had similar rates of STEMI presentation (66.3% vs 68.6%, SMD = 0.050). Types of tMCS were similar between patients with and without cancer, including IABP (89.4% vs 90.3%, SMD = 0.050), pLVAD (11.7% vs 11.1%, SMD = 0.018), and ECMO (1.9% vs 3.0%, SMD = 0.069). Patients with cancer were managed with PCI at a similar rate as those without cancer (59.0% vs 56.0%, SMD = 0.059) but were less likely to undergo CABG (21.2% vs 26.2%, SMD = 0.118). Admission characteristics prior to PSM are summarized in Supplemental Table S3.

After PSM, variables were adequately balanced between admissions with and without cancer, including STEMI presentation (66.1% vs 66.2%, SMD = 0.002), management with PCI (58.8% vs 58.7%, SMD = 0.002), and CABG (21.4% vs 21.0%, SMD = 0.009). After stratifying patients by cancer type, all variables remained balanced between admissions without cancer and admissions with solid cancer. However, admissions with hematologic cancer had increased rates of CABG (26.1% vs 21.0%, SMD = 0.118) and invasive hemodynamic monitoring (51.0% vs 44.2%, SMD = 0.137) and lower rate of Medicare or Medicaid insurance status (68.2% vs 73.0%, SMD = 0.106) compared with admissions without cancer. Admissions characteristics after PSM are summarized in Table 1 and stratified by cancer type in Supplemental Table S4.

**Table 1:** Baseline and hospitalization characteristics after propensity score matching.

	No cancer n = 12,870	Any cancer n = 1287	SMD
Age, y	69.4 ± 11.7	69.4 ± 11.3	0.001
Female sex	3688 (28.7)	364 (28.3)	0.008
Non-white race	3652 (28.4)	368 (28.6)	0.005
Comorbidities			
Prior MI	1019 (7.9)	106 (8.2)	0.012
Prior PCI	1008 (7.8)	98 (7.6)	0.008
Prior CABG	268 (2.1)	25 (1.9)	0.010
Heart failure	569 (4.4)	57 (4.4)	<0.001
Atrial fibrillation	3399 (26.4)	344 (26.7)	0.007
Anemia	3362 (26.1)	338 (26.3)	0.003
Chronic lung disease	3008 (23.4)	305 (23.7)	0.008
Smoking	3234 (25.1)	328 (25.5)	0.008
Diabetes	4039 (31.4)	405 (31.5)	0.002
Obesity	1171 (9.1)	121 (9.4)	0.010
Hypertension	6501 (50.5)	647 (50.3)	0.005
Liver disease	315 (2.4)	34 (2.6)	0.012
Peripheral vascular disease	1502 (11.7)	147 (11.4)	0.008
CKD	2683 (20.8)	270 (21.0)	0.003
Prior stroke	336 (2.6)	32 (2.5)	0.008
Prior VTE	246 (1.9)	29 (2.3)	0.024
Thrombocytopenia	1795 (13.9)	183 (14.2)	0.008
Hospitalization characteristics			
STEMI presentation	8520 (66.2)	851 (66.1)	0.002
Cardiac arrest	2018 (15.7)	201 (15.6)	0.002
Mechanical complication	180 (1.4)	19 (1.5)	0.012
MCS type			
IABP	11,522 (89.5)	1152 (89.5)	0.011
pLVAD	1492 (11.6)	149 (11.6)	0.001
ECMO	247 (1.9)	25 (1.9)	0.050
PCI	7557 (58.7)	757 (58.8)	0.002
CABG	2703 (21.0)	275 (21.4)	0.009
Invasive hemodynamic monitoring	5688 (44.2)	591 (45.9)	0.035
Mechanical ventilation	6567 (51.0)	654 (50.8)	0.026
Vasopressor used	1147 (8.9)	119 (9.2)	0.002
Palliative care	1044 (8.1)	105 (8.2)	0.054
DNR status	1128 (8.8)	115 (8.9)	0.005
Medicare or Medicaid	9392 (73.0)	935 (72.6)	0.054

Values are mean ± SD or n (%).

CABG, coronary artery bypass grafting; CKD, chronic kidney disease; DNR, do not resuscitate; ECMO, extracorporeal membrane oxygenation; IABP, intra-aortic balloon pump; MCS, mechanical circulatory support; MI, myocardial infarction; PCI, percutaneous coronary intervention; pLVAD, peripheral left ventricular assist device; SMD, standardized mean difference; STEMI, ST-elevation myocardial infarction; VTE, venous thromboembolism.

### Outcomes of patients with cancer compared with patients without cancer after PSM

**Figure 1:**
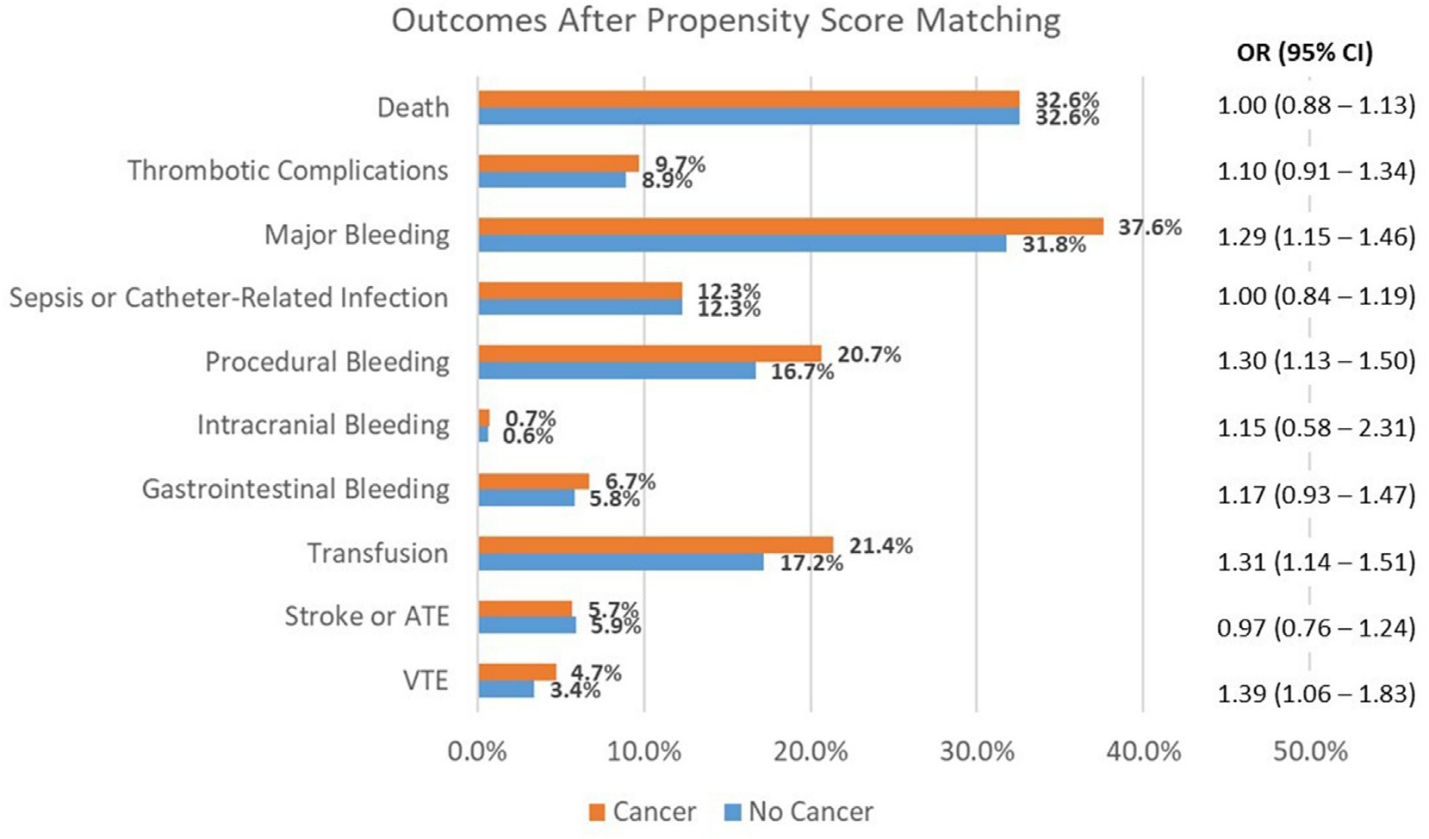
**Outcomes of patients hospitalized for acute myocardial infarction and cardiogenic shock (AMI–CS) managed with mechanical circulatory support (MCS) with vs without cancer.** Patient outcomes after propensity score matching of patients with vs without cancer hospitalized for AMI–CS managed with MCS. Logistic regression modeling used to estimate odds ratio (OR) and 95% CI of outcomes in patients with cancer compared with patients without cancer. ATE, aortic thromboembolism; VTE, venous thromboembolism.

After PSM, there was no significant difference in in-hospital mortality (OR, 1.00; 95% CI, 0.88-1.13) between admissions with and without cancer. Similarly, there was no statistically significant difference in rates of composite thrombotic complications (OR, 1.10; 95% CI, 0.91-1.34), though cancer was associated with higher risk of VTE (OR, 1.39; 95% CI, 1.06-1.83). Admissions with cancer had higher rates of major bleeding composite outcome (OR, 1.29, 95% CI, 1.15-1.46), procedure-related bleeding (OR, 1.30, 95% CI, 1.13-1.50) and transfusion of blood products (OR, 1.31, 95% CI, 1.14-1.51) compared with those without cancer. There was no difference in risk of sepsis or catheter-related infections (OR, 1.00; 95% CI, 0.84-1.19). Admission outcomes are summarized in Figure 1.

After stratifying by cancer type, admissions with solid cancer and hematologic cancer similarly were associated with increased rates of major bleeding, procedure-related bleeding, and transfusion, without significant difference in in-hospital death, thrombotic complications, sepsis, or catheter-related infections compared with those without cancer (Supplemental Table S5). However, after adjusting for variables that differed (SMD ≥ 0.10) between hematologic and noncancer admissions (management with CABG, invasive hemodynamic monitoring, and insurance status), patients with hematologic cancer did not have an increased rate of VTE (adjusted OR, 1.03; 95% CI, 0.63-1.66). Conversely, admissions with solid cancer did have an increased rate of VTE (OR, 1.62; 95% CI, 1.17-2.35). Logistic regression after stratification by cancer type and PSM is summarized in Supplemental Table S6.

### Risk factors for in-hospital death, thrombotic complications, and major bleeding in patients with cancer

Among unweighted admissions with cancer, 427 (32.7%), 128 (9.8%), and 490 (37.6%) had in-hospital death, thrombotic complication, and major bleeding, respectively. Admissions characteristics between patients with and without in-hospital death, thrombotic complications, and bleeding are summarized in Table 2. After multivariable logistic regression modeling, age, STEMI, cardiac arrest, pLVAD, ECMO, mechanical ventilation, palliative care, and DNR status were associated with increased risk of in-hospital death. Gynecologic cancer (OR, 0.18; 95% CI, 0.04-0.85), myeloproliferative neoplasms or myelodysplastic syndromes (OR, 0.50; 95% CI, 0.32-0.78), anemia, smoking, and CABG were associated with lower risks of in-hospital death.

**Table 2:** Comparison of unweighted admissions with death, bleeding, or thrombosis with patients without complications.

	No Bleeding n = 813	Bleeding n = 491	*P* Value	No Thrombosis n = 1176	Thrombosis n = 128	*P* Value	No Death n = 874	Death n = 427	*P* Value
Age, y	69.0 ± 11.3	70.3 ± 11.4	.039	69.6 ± 11.4	68.4 ± 10.6	.21	68.4 ± 11.6	71.8 ± 10.5	<.001
Female sex	229 (28.2)	139 (28.3)	1.00	326 (27.7)	42 (32.8)	.25	250 (28.6)	118 (27.6)	.74
Non-White race	220 (27.1)	148 (30.1)	.25	333 (28.3)	35 (27.3)	.92	249 (28.5)	117 (27.4)	.69
Cancer characteristics									
Solid cancer	508 (63.3)	289 (59.7)	.21	720 (62.0)	77 (61.6)	.92	522 (60.1)	275 (65.6)	.058
Hematologic cancer	310 (38.1)	204 (41.5)	.24	466 (39.6)	48 (37.5)	.70	361 (41.3)	152 (35.6)	.053
Colorectal	37 (4.5)	31 (6.3)	.20	61 (5.2)	7 (5.5)	.83	48 (5.5)	20 (4.7)	.60
Upper GI cancer	11 (1.3)	14 (2.8)	.063	20 (1.7)	5 (3.9)	.090	18 (2.1)	7 (1.6)	.67
Nonluminal GI cancer	16 (2.0)	7 (1.4)	.52	21 (1.8)	2 (1.6)	1.00	14 (1.6)	9 (2.1)	.51
Lung	118 (14.5)	58 (11.8)	.18	159 (13.5)	17 (13.3)	1.00	101 (11.6)	75 (17.6)	.004
Breast	21 (2.6)	16 (3.3)	.49	34 (2.9)	3 (2.3)	1.00	27 (3.1)	10 (2.3)	.48
Prostate	93 (11.4)	49 (10.0)	.46	136 (11.6)	6 (4.7)	.016	90 (10.3)	52 (12.2)	.34
Gynecologic cancer	16 (2.0)	8 (1.6)	.83	21 (1.8)	3 (2.3)	.72	22 (2.5)	2 (0.5)	.008
Urogenital	40 (4.9)	18 (3.7)	.33	55 (4.7)	3 (2.3)	.36	40 (4.6)	18 (4.2)	.89
Any leukemia	63 (7.7)	48 (9.8)	.22	105 (8.9)	6 (4.7)	.13	72 (8.2)	38 (8.9)	.67
Lymphoma	124 (15.2)	71 (14.5)	.75	171 (14.5)	24 (18.7)	.24	129 (14.8)	66 (15.5)	.74
Plasma cell	28 (3.4)	19 (3.9)	.76	41 (3.5)	6 (4.7)	.45	29 (3.3)	18 (4.2)	.43
Lymphoid leukemia	38 (4.7)	35 (7.1)	.081	69 (5.9)	4 (3.1)	.31	43 (4.9)	29 (6.8)	.20
Myeloid leukemia	25 (3.1)	13 (2.6)	.74	3 (3.1)	2 (1.6)	.58	29 (3.3)	9 (2.1)	.29
MDS/MPN	135 (16.6)	89 (18.1)	.50	206 (17.5)	18 (14.1)	.39	174 (19.9)	50 (11.7)	<.001
Metastatic cancer	181 (22.3)	101 (20.6)	.49	255 (21.7)	27 (21.1)	1.00	176 (20.1)	105 (24.6)	.073
Brain primary or metastasis	20 (2.5)	8 (1.6)	.43	22 (1.9)	6 (4.7)	.049	15 (1.7)	12 (2.8)	.21
Comorbidities									
Prior MI	64 (7.9)	44 (9.0)	.53	100 (8.5)	8 (6.2)	.50	80 (9.1)	28 (6.6)	.13
Prior PCI	71 (8.7)	31 (6.3)	.14	94 (8.0)	8 (6.2)	.60	69 (7.9)	33 (7.7)	1.00
Prior CABG	20 (2.5)	5 (1.0)	.093	25 (2.1)	0	.16	16 (1.8)	9 (2.1)	.83
Heart failure	39 (3.6)	29 (5.9)	.053	48 (4.1)	10 (7.8)	.067	39 (4.5)	19 (4.4)	1.00
Anemia	203 (25.0)	147 (29.9)	.053	311 (26.4)	39 (30.5)	.34	254 (29.1)	94 (22.0)	.008
Chronic lung disease	199 (24.5)	112 (22.8)	.50	284 (24.1)	27 (21.1)	.51	211 (24.1)	100 (23.4)	.84
Smoking	237 (29.1)	92 (18.7)	<.001	301 (25.6)	28 (21.9)	.39	235 (26.9)	94 (22.0)	.067
Diabetes	244 (30.0)	167 (34.0)	.14	370 (31.5)	41 (32.0)	.92	280 (32.0)	129 (30.2)	.52
Obesity	72 (8.9)	50 (10.2)	.43	102 (8.7)	20 (15.6)	.016	85 (9.7)	36 (8.4)	.48
Hypertension	422 (51.9)	234 (47.7)	.14	588 (50.0)	68 (53.1)	.52	439 (50.2)	215 (50.3)	1.00
Liver disease	18 (2.2)	17 (3.5)	.21	32 (2.7)	3 (2.3)	1.00	26 (3.0)	9 (2.1)	.47
Peripheral vascular disease	71 (8.7)	76 (15.5)	<.001	121 (10.3)	26 (20.3)	<.001	98 (11.2)	49 (11.5)	.93
CKD	157 (19.3)	116 (23.6)	.068	243 (20.7)	30 (23.4)	.49	181 (20.7)	92 (21.5)	.77
Prior stroke	20 (2.5)	12 (2.4)	1.00	27 (2.3)	5 (3.9)	.23	22 (2.5)	10 (2.3)	1.00
Prior VTE	23 (2.8)	14 (2.8)	1.00	35 (3.0)	2 (1.6)	.57	29 (3.3)	8 (1.9)	.16
Thrombocytopenia	91 (11.2)	95 (19.4)	<.001	165 (14.0)	21 (16.4)	.50	133 (15.2)	53 (12.4)	.21
Hospitalization characteristics									
STEMI presentation	582 (71.6)	282 (57.4)	<.001	787 (66.9)	77 (60.2)	.14	554 (63.4)	307 (71.9)	.002
Cardiac arrest	138 (17.0)	63 (12.8)	.048	181 (15.4)	20 (15.6)	.90	99 (11.3)	101 (23.6)	<.001
Mechanical complications	12 (1.5)	7 (1.4)	1.00	18 (1.5)	1 (0.8)	1.00	12 (1.4)	7 (1.6)	.81
MCS type									
IABP	741 (91.1)	425 (86.6)	.012	1053 (89.5)	113 (88.3)	.65	802 (91.8)	362 (84.8)	<.001
pLVAD	77 (9.5)	75 (15.3)	.002	134 (11.4)	18 (14.1)	.38	79 (9.0)	72 (16.9)	<.001
ECMO	9 (1.1)	16 (3.3)	.011	21 (1.8)	4 (3.1)	.30	11 (1.3)	14 (3.3)	.017
PCI	542 (66.7)	227 (46.2)	<.001	703 (59.8)	66 (51.6)	.088	505 (57.8)	261 (61.1)	.25
CABG	94 (11.6)	182 (37.1)	<.001	235 (20.0)	41 (31.2)	.003	223 (25.5)	53 (12.4)	<.001
Invasive hemodynamic monitoring	370 (45.5)	234 (47.7)	.46	542 (46.1)	62 (48.4)	.64	401 (45.9)	203 (47.5)	.59
Mechanical ventilation	399 (49.1)	266 (54.2)	.077	591 (50.3)	74 (57.8)	.11	373 (42.7)	289 (67.7)	<.001
Vasopressor used	72 (8.9)	49 (10.0)	.49	112 (9.5)	9 (7.0)	.42	77 (8.8)	44 (10.3)	.42
Palliative care	69 (8.5)	44 (9.0)	.76	101 (8.6)	12 (9.4)	.74	26 (3.0)	87 (20.4)	<.001
DNR status	76 (9.3)	47 (9.6)	.92	115 (9.8)	8 (6.2)	.26	36 (4.1)	87 (20.4)	<.001
Medicare or Medicaid	579 (71.2)	373 (76.0)	.062	858 (73.0)	94 (73.4)	1.00	613 (70.1)	336 (78.7)	.001
Private insurance	192 (23.6)	94 (19.1)	.062	259 (22.0)	27 (21.1)	.91	211 (24.1)	75 (17.6)	.007
Complications									
Post-procedure bleeding	N/A	N/A	N/A	223 (19.2)	44 (35.2)	<.001	194 (22.3)	73 (17.4)	.047
Intracranial bleed	N/A	N/A	N/A	5 (0.4)	4 (3.2)	.007	4 (0.5)	5 (1.2)	.14
Gastrointestinal bleed	N/A	N/A	N/A	80 (6.9)	6 (4.8)	.45	57 (6.6)	29 (6.9)	.81
Transfusion	N/A	N/A	N/A	234 (20.1)	41 (32.8)	.002	188 (21.7)	87 (20.8)	.77
Stroke or arterial thromboembolism	35 (4.4)	39 (8.1)	.007	N/A	N/A	N/A	57 (6.6)	17 (4.1)	.074
VTE	22 (2.7)	38 (7.8)	<.001	N/A	N/A	N/A	43 (4.9)	17 (4.1)	.57

Values are mean ± SD or n (%).

CABG, coronary artery bypass grafting; CKD, chronic kidney disease; DNR, do not resuscitate; ECMO, extracorporeal membrane oxygenation; GI, gastrointestinal; IABP, intra-aortic balloon pump; MCS, mechanical circulatory support; MDS, myelodysplastic syndrome; MI, myocardial infarction; MPN, myeloproliferative neoplasm; N/A, not available; PCI, percutaneous coronary intervention; pLVAD, peripheral left ventricular assist device; STEMI, ST-elevation myocardial infarction; VTE, venous thromboembolism.

**Figure 2:**
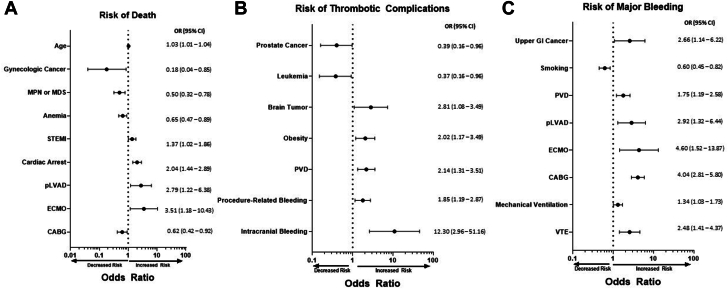
**Risk factors for in-hospital death, thrombotic complications, and major bleeding among patients with cancer.** Forest plots of risk factors for (**A**) in-hospital death, (**B**) thrombotic complications, (**C**) and major bleeding. CABG, coronary artery bypass grafting; ECMO, extracorporeal membrane oxygenation; GI, gastrointestinal; MDS, myelodysplastic syndrome; MPN, myeloproliferative neoplasm; STEMI, ST-elevation myocardial infarction; pLVAD, peripheral left ventricular assist device; PVD, peripheral vascular disease; VTE, venous thromboembolism.

After multivariable logistic regression, brain primary tumor or metastasis (OR, 2.81; 95% CI, 1.08-7.32), obesity, peripheral vascular disease, and intracranial bleeding were associated with increased risk of thrombotic complication. Prostate cancer (OR, 0.39; 95% CI, 0.16-0.96) and leukemia (OR, 0.37; 95% CI, 0.16-0.96) were associated with lower risk of thrombotic complication. Upper gastrointestinal cancer (OR, 2.66; 95% CI, 1.14-6.22), peripheral vascular disease, pLVAD, ECMO, CABG, mechanical ventilation, and VTE were associated with increased risk of major bleeding (Figure 2).

### Trends in cancer patients admitted for AMI–CS and managed with MCS and types of MCS use

**Figure 3:**
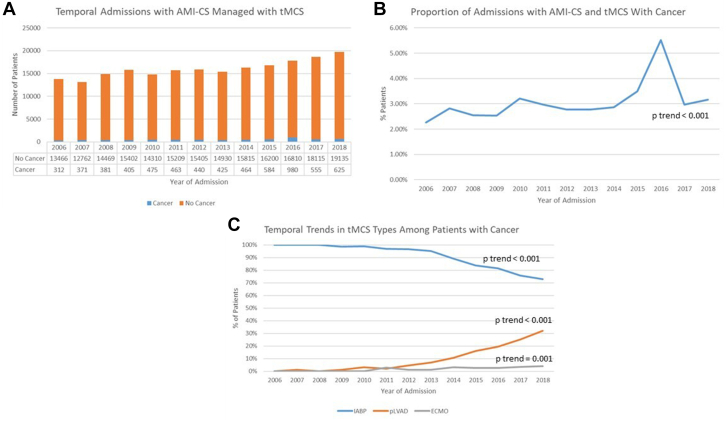
**Temporal trends of patients with cancer and AMI–CS managed with MCS.** (**A**) Bar graph depicting number of admissions of AMI–CS managed with MCS with and without cancer. (**B**) There has been a temporal increase in the proportion of patients with AMI–CS managed with MCS with cancer. (**C**) Among patients with cancer, there has a been a temporal trend in decreased IABP use and increased pLVAD and ECMO use. AMI, acute myocardial infarction; CS, cardiogenic shock; ECMO, extracorporeal membrane oxygenation; IABP, intra-aortic balloon pump; MCS, mechanical circulatory support; pLVAD, peripheral left ventricular assist device; tMCS, temporary mechanical circulatory support.

From 2006 to 2018, the number of weighted admissions for AMI–CS managed with tMCS increased from 13,466 to 19,135 among admissions without cancer and 312 to 625 among those with cancer (*P*_trend_ < .001 for both). The proportion of admissions with cancer increased from 2.27% in 2006 to 3.16% in 2018 (*P*_trend_ < .001). Among admissions with cancer with AMI–CS and managed with MCS, the proportion of admissions managed with IABP decreased from 100% in 2006 to 72.8% in 2018 (*P*_trend_ < .001). Admissions managed with pLVAD increased from 0% in 2006 to 32.0% in 2018 (*P*_trend_ < .001). Similarly, admissions managed with ECMO increased from 0% in 2006 to 4.0% in 2018 (*P*_trend_ = .001). Temporal trends of number of admissions admitted with AMI–CS managed with MCS, proportion of admissions with cancer, and types of MCS used in patients with cancer are shown in Figure 3.

### Predictors of tMCS use among admissions with cancer

Among admissions with AMI–CS and cancer, those managed with tMCS were younger (mean 69.5 vs 73.3 years, *P* < .001) and less likely to be female (28.2% vs 45.7%, *P* < .001). Admissions managed with MCS were also less likely to have solid cancer, metastatic disease, nonluminal gastrointestinal cancer, palliative care consult, DNR status, and Medicare or Medicaid insurance (Table 3). After multivariable logistic regression, age, female sex, nonluminal gastrointestinal cancer, chronic lung disease, peripheral vascular disease, CKD, prior stroke, and DNR status were associated with decreased MCS use. Mechanical complication, STEMI, PCI, CABG, invasive hemodynamic monitoring, and mechanical ventilation were associated with increased MCS use (Table 4).

**Table 3:** Characteristics of cancer admissions with AMI–CS managed with vs without MCS.

	No MCS n = 2347	MCS n = 1304	*P* Value
Age, y	73.3 ± 11.6	69.5 ± 11.3	<.001
Female sex	838 (45.7)	368 (28.2)	<.001
Non-white race	647 (27.6)	368 (28.2)	.67
Cancer characteristics			
Solid cancer	1557 (66.3)	811 (62.2)	.013
Hematologic cancer	822 (35.0)	514 (39.4)	.009
Colorectal	117 (5.0)	68 (5.2)	.75
Upper GI cancer	46 (2.0)	25 (1.9)	1.00
Nonluminal GI cancer	103 (4.4)	23 (1.8)	<.001
Lung	418 (17.8)	176 (13.5)	.001
Breast	95 (4.0)	37 (2.8)	.064
Prostate	216 (9.2)	142 (10.9)	.10
Gynecologic cancer	44 (1.9)	24 (1.8)	1.00
Urogenital	121 (5.2)	58 (4.4)	.38
Any leukemia	224 (9.5)	111 (8.5)	.31
Lymphoma	319 (13.6)	195 (14.9)	.27
Plasma cell	104 (4.4)	47 (3.6)	.26
Lymphoid leukemia	150 (6.4)	73 (5.6)	.35
Myeloid leukemia	74 (3.1)	38 (2.9)	.76
MDS/MPN	311 (13.2)	224 (17.2)	.001
Metastatic cancer	613 (26.1)	282 (21.6)	.003
Brain primary or metastasis	64 (2.7)	28 (2.1)	.32
Comorbidities			
Prior MI	192 (8.2)	108 (8.3)	.95
Prior PCI	201 (8.6)	102 (7.8)	.45
Prior CABG	129 (5.5)	25 (1.9)	<.001
Heart failure	70 (3.0)	58 (4.4)	.024
Atrial fibrillation	686 (29.2)	350 (26.8)	.126
Anemia	731 (31.1)	350 (26.8)	.006
Chronic lung disease	675 (28.8)	311 (23.8)	.001
Smoking	589 (25.1)	329 (25.2)	.94
Diabetes	709 (30.2)	411 (31.5)	.41
Obesity	175 (7.5)	122 (9.4)	.050
Hypertension	1194 (50.9)	656 (50.3)	.76
Liver disease	66 (2.8)	35 (2.7)	.92
Peripheral vascular disease	376 (16.0)	147 (11.3)	<.001
CKD	665 (28.3)	273 (20.9)	<.001
Prior stroke	83 (3.5)	32 (2.5)	.076
Prior VTE	76 (3.2)	37 (2.8)	.55
Hospitalization characteristics			
STEMI presentation	1190 (50.7)	864 (66.3)	<.001
Cardiac arrest	321 (13.7)	201 (15.4)	.15
Mechanical complication	8 (0.3)	19 (1.5)	<.001
PCI	678 (28.9)	769 (59.0)	<.001
CABG	160 (6.8)	276 (21.1)	<.001
Invasive hemodynamic monitoring	550 (23.4)	604 (46.3)	<.001
Mechanical ventilation	917 (39.1)	665 (51.0)	<.001
Vasopressor used	229 (9.8)	121 (9.3)	.64
Palliative care	354 (15.1)	113 (8.7)	<.001
DNR status	417 (17.8)	123 (9.4)	<.001
Medicare or Medicaid	1864 (79.4)	952 (73.0)	<.001

Values are mean ± SD or n (%).

AMI, acute myocardial infarction; CABG, coronary artery bypass grafting; CKD, chronic kidney disease; CS, cardiogenic shock; DNR, do not resuscitate; GI, gastrointestinal; MCS, mechanical circulatory support; MDS, myelodysplastic syndrome; MI, myocardial infarction; MPN, myeloproliferative neoplasm; PCI, percutaneous coronary intervention; SD, standard deviation; STEMI, ST-segment myocardial infarction; VTE, venous thromboembolism.

**Table 4:** Predictors of MCS use among admissions with cancer and AMI–CS.

	OR (95% CI)
Age	0.99 (0.98-0.99)
Female sex	0.75 (0.62-0.89)
Chronic lung disease	0.79 (0.66-0.95)
Peripheral vascular disease	0.68 (0.53-0.85)
Chronic kidney disease	0.80 (0.66-0.97)
Prior stroke	0.57 (0.36-0.92)
STEMI	1.66 (1.40-1.97)
Mechanical complication	4.14 (1.64-10.46)
PCI	4.20 (3.52-5.01)
CABG	7.25 (5.62-9.35)
Invasive hemodynamics	1.97 (1.67-2.33)
Mechanical ventilation	1.69 (1.43-1.99)
DNR status	0.71 (0.54-–0.93)
Nonluminal GI cancer	0.42 (0.25-–0.70)

AMI, acute myocardial infarction; CABG, coronary artery bypass grafting; CS, cardiogenic shock; DNR, do not resuscitate; GI, gastrointestinal; MCS, mechanical circulatory support; OR, odds ratio; PCI, percutaneous coronary intervention; STEMI, ST-segment myocardial infarction.

## Discussion

Cardiovascular disease, including AMI and AMI–CS, are increasingly recognized complications of cancer and cancer therapy. Patients with AMI–CS are often managed with tMCS, including patients with cancer. Our study suggests that among admissions with AMI–CS managed with tMCS, cancer was not associated with higher risk of in-hospital mortality. However, cancer was associated with increased risk of major bleeding, procedure-related bleeding, transfusion, and VTE. Additionally, our study identified potential risk factors for in-hospital death (STEMI, non-IABP MCS, cardiac arrest), major bleeding (upper gastrointestinal cancer, peripheral vascular disease, ECMO, VTE), and thrombotic complications (brain primary tumor or metastasis) among patients with cancer and AMI–CS managed with MCS. Our study also demonstrated an increasing prevalence of patients with AMI–CS managed with MCS with cancer, stressing the importance of investigating outcomes and risk factors in this patient population (Central Illustration). We also examined patient factors associated with tMCS use in patients with cancer, and our findings suggest that age, female sex, DNR status, and nonluminal gastrointestinal cancer were associated with decreased use of tMCS and STEMI, while mechanical complications and revascularization were associated with increased use.

**Central Illustration:**
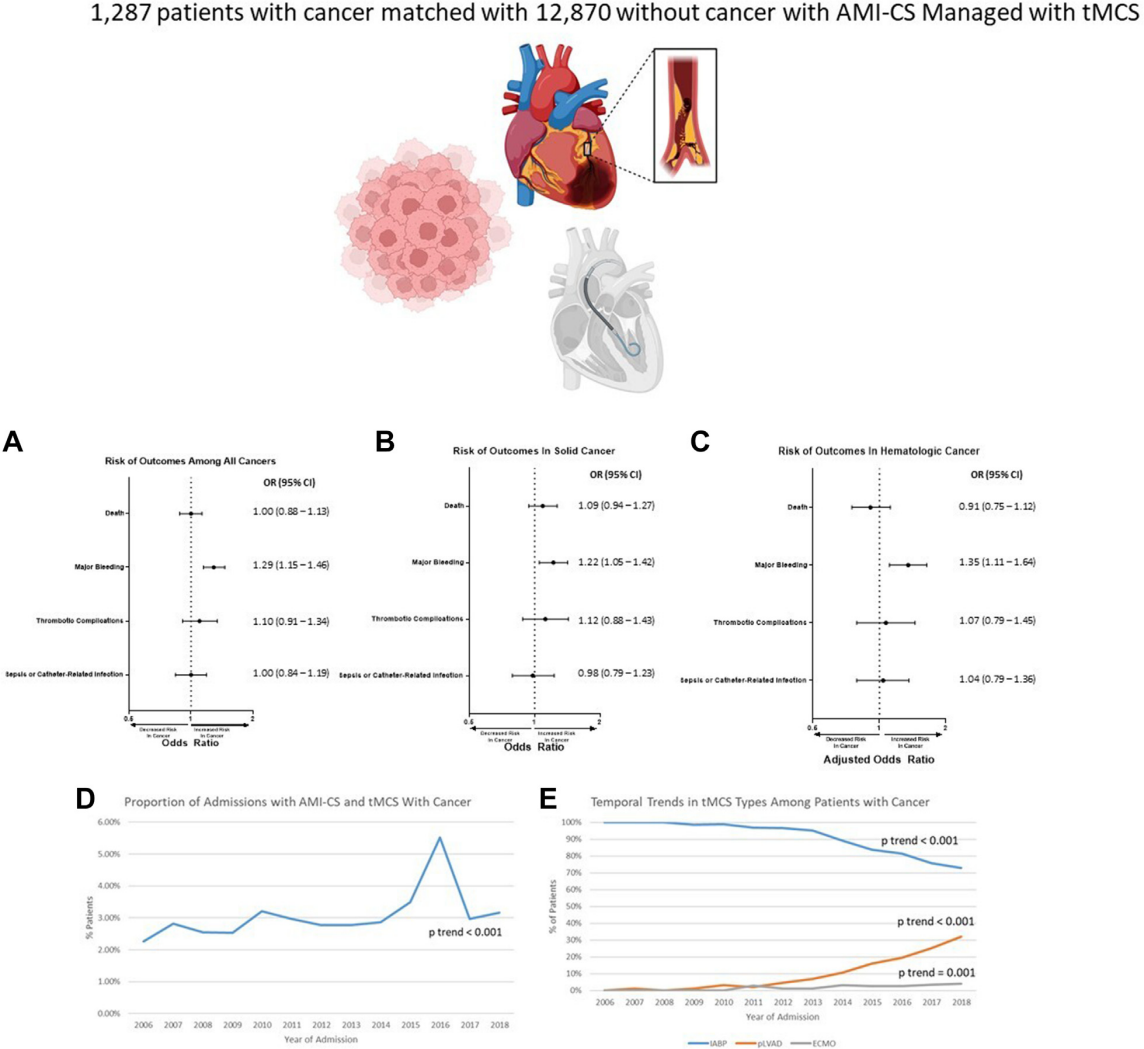
In-hospital outcomes and trends among patients with cancer with AMI–CS managed with tMCS. (**A**) Among patients admitted for AMI–CS managed with MCS, patients with cancer were associated with increased risk of major bleeding but not in-hospital death or thrombotic complications. After stratifying for solid cancer (**B**) and hematologic cancer (**C**), patients with cancer were associated with increased risk of major bleeding but not death, thrombotic complications, sepsis, or catheter-related infections. (**D**) There was and increased temporal trend of prevalence of patients with cancer among patients admitted for AMI–CS managed with MCS. (**E**) Among patients with cancer, there was a temporal trend of decreased IABP use and increase in pLVAD and ECMO use. AMI, acute myocardial infarction; CS, cardiogenic shock; ECMO, extracorporeal membrane oxygenation; IABP, intra-aortic balloon pump; MCS, mechanical circulatory support; OR, odds ratio; tMCS, temporary mechanical circulatory support.

The successful use of MCS in patients with active cancer has been described in case reports as a bridge to recovery in a variety of disease states unique to cardio-oncology; examples include immune checkpoint inhibitor myocarditis, pulmonary tumor thrombotic microangiopathy, obstructive shock due to tumor compression, and anthracycline-induced cardiomyopathy.^18^, ^19^, ^20^, ^21^ However, the most common cause of CS in patients with cancer is AMI, and data on outcomes of patients with cancer with AMI–CS managed with MCS are sparse.^22^ Our study findings suggest that patients with cancer have similar in-hospital mortality compared with patients without cancer. Nonetheless, a substantial proportion of patients with cancer in this analysis had metastatic disease (21.6%). This suggests that cancer, even metastatic cancer, should not be considered an absolute contraindication for MCS in AMI–CS. Additionally, infection is a feared complication among patients with cancer given immunosuppression due to cancer-related therapy, immune and bone marrow dysfunction due to cancer itself, and indwelling catheters. However, among patients with AMI–CS managed with tMCS, cancer was not associated with increased risk of sepsis or catheter-related infections.

In our study, cancer was associated with increased risk of major bleeding including postprocedural bleeding and transfusion, consistent with prior studies of patients managed with percutaneous MCS^23^ and durable LVAD.^24^ There is a known risk of bleeding complications for patients managed with tMCS among the general population that is related to vascular access complications, anticoagulation and antiplatelet use, and coagulation factor and platelet consumption due to devices.^25^, ^26^, ^27^ These risk factors are exacerbated among patients with cancer in addition to cancer-specific risk factors for bleeding (mucosal friability due to chemotherapy or tumor infiltration, and chronic disseminated intravascular coagulation).^28^, ^29^, ^30^, ^31^ Further investigation is needed to mitigate bleeding risk in this patient population.

Our study identified potential risk factors for bleeding complications among patients with cancer and AMI–CS managed with MCS. Upper gastrointestinal cancers were associated with increased risk of bleeding in our cohort. This is consistent with prior studies suggesting luminal gastrointestinal cancers are associated with increased risk of bleeding among patients managed with MCS and receiving anticoagulation.^23^^,^^32^ Additionally, ECMO and pLVAD were associated with increased bleeding compared with IABP. This has also previously been demonstrated and may be due to increased platelet shearing from device-related hemolysis and larger bore vascular access.^33^^,^^34^ Peripheral arterial disease was another risk factor for bleeding among patients with cancer that has also been noted among the general population managed with MCS.^35^

Thrombotic complications are a known risk among patients managed with MCS as well as patients with cancer.^36^, ^37^, ^38^ In our study, there was no association between cancer and thrombotic complications, aside from an increased risk of VTE. Additionally, our study identified potential risk factors for thrombosis. Cancer-specific risk factors for thrombotic complications in our study were the presence of primary brain tumor or metastasis. Patients with primary brain tumors have been noted to be at high risk of VTE due to increased production of tissue factor, a potent procoagulant. Prior studies have noted a prevalence of VTE of upwards of 30% among patients with primary brain tumors and 20% in patients with brain metastasis.^39^ Interestingly, our study did not find an association between brain tumors and intracranial bleeding. This is in line with prior studies suggesting that anticoagulation in patients with brain tumors does not increase risk of intracranial hemorrhage.^40^

Our study examined factors associated with tMCS use in patients with cancer, and our findings suggest that age, female sex, peripheral vascular disease, CKD, prior stroke, DNR status, and nonluminal gastrointestinal cancer were associated with lower tMCS use. Prior studies in the general population have also suggested that older patients and female patients with CS (both AMI–CS and non-AMI–CS) are less likely to be managed with tMCS.^41^^,^^42^ Similarly, peripheral vascular disease and CKD have been associated with lower rates of tMCS use and may be related to increased vascular complications and in-hospital mortality.^43^ The association between nonluminal gastrointestinal cancer (including pancreatic and hepatobiliary) may be due to increased short-term mortality in this population compared to other cancer types, though further investigation is needed.^44^

### Limitations and considerations

This study has several limitations to consider. The first limitation is the retrospective, cross-sectional nature of our study, which makes it prone to residual, unmeasured confounding. The data in the NIS are abstracted from ICD-9 and ICD-10 billing codes, which are prone to misclassification. Data on cancer activity, treatment, laboratory values including blood counts, duration of disease, and genetic testing are not reported and may affect outcomes in this patient population. Additionally, patients with and without cancer were selected by clinicians to be managed with tMCS, and thus, there may be selection and treatment bias. The NIS does not provide the temporal relationship of diagnoses, therefore thrombotic and bleeding outcomes in our cohort may have occurred prior to hospitalization or implantation of MCS. Further, the NIS is restricted to the hospitalization episode, so no postdischarge data is available. Granular data on details of revascularization (ie, disease severity and vessels revascularized) and antithrombotic management are not reported in the database. Additionally, we did not correct for multiple comparisons, and therefore, there is an increased risk of type I error.

## Conclusions

Among patients with AMI–CS managed with tMCS, there was no association between cancer and in-hospital mortality after propensity matching. Cancer was associated with increased risk of major bleeding and VTE. Our study suggests that among patients with cancer, MCS may provide benefit, with risks that are similar to the general population. Therefore, clinicians managing AMI–CS should not consider cancer a contraindication for management with tMCS even in the presence of metastatic disease. However, clinicians should be aware of increased thrombotic risk among patients with cancer with brain tumors, obesity, and peripheral vascular disease and of increased bleeding risk among patients with upper gastrointestinal cancer. Our results are hypothesis-generating given the retrospective nature of the study. Future prospective studies and trials of tMCS in AMI–CS should include patients with cancer in order to confirm our findings and better delineate risks and benefits of tMCS in this patient population.
