# Supplementary material for: Outcomes of Patients With Cancer With Myocardial Infarction-Associated Cardiogenic Shock Managed With Mechanical Circulatory Support
Source: J Soc Cardiovasc Angiogr Interv. 2023 Nov 13;3(3Part A):101208. doi: 10.1016/j.jscai.2023.101208 (PMC11307771; doi:10.1016/j.jscai.2023.101208)
Supplement: Supplemental Tables S1-S6 [file mmc1.docx]

**Outcomes of Patients with Cancer Admitted with Myocardial Infarction and Cardiogenic Shock Managed with Mechanical Circulatory Support**

Supplemental Table 1: Co-Morbidity and Outcomes ICD-9 and ICD-10 Codes, Pages 2 – 6

Supplemental Table 2: Cancer type ICD-9 and ICD-10 codes, Page 6

Supplemental Table 3: Patient Characteristics Prior to Propensity Score Matching, Page 7

Supplemental Table 4: Patient and Hospitalization Characteristics After Propensity Score Matching Stratified by Cancer Type, Pages 8 – 9

Supplemental Table 5: Outcomes of Patients with versus without Cancer After PSM and Stratification by Cancer Type, Pages 9 – 10

Supplemental Table 6: Logistic Regression of Outcomes Stratified by Cancer Type after PSM, Pages 10 - 11

**Supplemental Table 1: Co-Morbidity and Outcomes ICD-9 and ICD-10 Codes**

|  | ICD-9 Codes | ICD-10 Codes |
| --- | --- | --- |
| NSTEMI | 410.7x | I21.4 |
| STEMI | 410.x (except 410.7x) | I21.x (except I21.4) |
| Thrombocytopenia | 287.49, 287.5 | D69.59, D69.6 |
| Cardiogenic Shock | 78551, 99801 | R57, T8111XA |
| Smoking | 305.1, V15.82 | F17.*, Z87.891 |
| Prior myocardial infraction | 412 | I25.2 |
| Prior PCI | V45.82 | Z95.5, Z9861 |
| Prior CABG | V45.81 | Z95.1 |
| Congestive Heart Failure | AHRQ comorbidity variable included in database | AHRQ comorbidity variable included in database |
| Anemia | AHRQ comorbidity variable included in database | AHRQ comorbidity variable included in database |
| Chronic Lung Disease | AHRQ comorbidity variable included in database | AHRQ comorbidity variable included in database |
| Diabetes | AHRQ comorbidity variable included in database | AHRQ comorbidity variable included in database |
| Liver disease | AHRQ comorbidity variable included in database | AHRQ comorbidity variable included in database |
| Peripheral vascular disease | AHRQ comorbidity variable included in database | AHRQ comorbidity variable included in database |
| Chronic kidney disease (renal failure) | AHRQ comorbidity variable included in database | AHRQ comorbidity variable included in database |
| Solid tumor with metastasis | AHRQ comorbidity variable included in database | AHRQ comorbidity variable included in database |
| Acquired immunodeficiency syndrome | AHRQ comorbidity variable included in database | AHRQ comorbidity variable included in database |
| Atrial Fibrillation | 423.* | I48.* |
| Prior Stroke | V125.4 | Z8673 |
| Cardiac Arrest | 427.5 | I46.2, I46.8, I46.9 |
| DNR Status | V49.86 | Z66 |
| Palliative Care | V66.7 | Z51.5 |
| Mechanical Complication | 429.5, 429.6, 429.71, 745.4, 429.79 | I51.1, I51.2, I51.0, Q21.0, I23.2, I23.3, I23.0 |
|  |  |  |
| Procedures |  |  |
| Percutaneous Coronary Intervention | 3601, 3602, 3605, 3606, 3607 | 0270346, 027034Z, 0270356, 027035Z, 027035Z, 0270366, 027036Z, 0270376, 0270376, 027037Z, 02703D6, 02703DZ, 02703E6, 02703EZ, 02703F6, 02703FZ, 02703G6, 02703GZ, 02703Z6, 02703ZZ, 0270446, 027044Z, 0270456, 027045Z, 027045Z, 0270466, 027046Z, 0270476, 027047Z, 02704D6, 02704DZ, 02704E6, 02704EZ, 02704F6, 02704FZ, 02704G6, 02704GZ, 02704Z6, 02704ZZ, 0271346, 027134Z, 0271356, 027135Z, 0271366, 027136Z, 0271376, 027137Z, 02713D6, 02713DZ, 02713E6, 02713EZ, 02713F6, 02713FZ, 02713G6, 02713GZ, 02713Z6, 02713ZZ, 0271446, 027144Z, 0271456, 027145Z, 0271466, 027146Z, 0271476, 027147Z, 02714D6, 02714DZ, 02714E6, 02714EZ, 02714F6, 02714FZ, 02714G6, 02714GZ, 02714Z6, 02714ZZ, 0272346, 027234Z, 0272356, 027235Z, 0272366, 027236Z, 0272376, 027237Z, 02723D6, 02723DZ, 02723E6, 02723EZ, 02723F6, 02723FZ, 02723G6, 02723GZ, 02723Z6, 02723ZZ, 0272446, 027244Z, 0272456, 027245Z, 027246Z, 0272476, 027247Z, 02724D6, 02724DZ, 02724E6, 02724EZ, 02724F6, 02724FZ, 02724G6, 02724GZ, 02724Z6, 02724ZZ, 0273346, 027334Z, 0273356, 027335Z, 0273366, 027336Z, 0273376, 027337Z, 02733D6, 02733DZ, 02733E6, 02733EZ, 02733F6, 02733FZ, 02733G6, 02733GZ, 02733Z6, 02733ZZ, 0273446, 027344Z, 0273456, 027345Z, 0273466, 027346Z, 0273476, 027347Z, 02734D6, 02734DZ, 02734E6, 02734EZ, 02734F6, 02734G6, 02734GZ, 02734Z6, 02734ZZ |
| Coronary Artery Bypass Grafting | 3610, 3611, 3612, 3612, 3613, 3614, 3615, 3616, 3617, 3619 | 0210093, 0210099, 021009C, 021009F, 021009W, 02100A3, 02100A8, 02100A9, 02100AC, 02100AF, 02100AW, 02100J3, 02100K9, 02100KC, 02100KF, 02100KW, 02100Z3, 02100Z8, 02100Z9, 02100ZC, 02100ZF, 0211093, 0211098, 0211099, 021109C, 021109F, 021109W, 02110A3, 02110A8, 02110A9, 02110AC, 02110AF, 02110AW, 02110J3, 02110J8, 02110J9, 02110JC, 02110JF, 02110JW, 02110K3, 02110K8, 02110K9, 02110KC, 02110KF, 02110KW, 02110Z3, 02110Z8, 02110Z9, 02110ZC, 02110ZF, 0212093, 0212098, 0212099, 021209C, 021209F, 021209W, 02120A3, 02120A8, 02120A9, 02120AC, 02120AF, 02120AW, 02120J3, 02120J8, 02120J9, 02120JC, 02120JF, 02120JW, 02120K3, 02120K8, 02120K9, 02120KC, 02120KF, 02120KW, 02120Z3, 02120Z8, 02120Z9, 02120ZC, 02120ZF, 0213093, 0213098, 0213099, 021309C, 021309F, 021309W, 02130A3, 02130A8, 02130A9, 02130AC, 02130AF, 2130AW, 02130J3, 02130J8, 02130J9, 02130JC, 02130JF, 02130JW, 02130K3, 02130K8, 02130K9, 02130KC, 02130KF, 02130KW, 02130Z3, 02130Z8, 02130Z9, 02130ZC, 02130ZF, 0210083, 0210088, 0210089, 021008C, 021008F, 021008W, 0210098, 02100J8, 02100J9, 02100JC, 02100JF, 02100JW, 02100K3, 02100K8, 0211083, 0211088, 0211089, 021108C, 021108F, 021108W, 0212083,0212088, 0212089, 021208C, 021208F, 021208W, 0213083, 0213088, 0213089, 021308C, 021308F, 021308W |
| Left Heart Catheterization | 3722, 8855, 8856, 8853 | 4A023N7, B21* |
| Invasive Hemodynamic Monitoring | 89.59, 89.64, 89.63, 37.21, 37.23 | 4A1239Z, 4A0239Z, 4A023N6, 4A023N8, 4A033J3, 4A033B3, 4A03353, 4A13353, 4A133B3, 4A133J3, 02HP32Z |
| Transfusion | 990, 9900, 9901, 9902, 9903, 9904, 9905, 9906, 9907, 9908, 9909 | 30230H0, 30230H1, 30230K0, 30230K1, 30230L1, 30230M0, 30230M1, 30230N0, 30230N1, 30230P0, 30230P1, 30230J0, 30230J1, 30230R0, 30230R1, 30230W0, 30230W1, 30233H0, 30233H1, 30233J0, 30233J1, 30233K0, 30233K1, 30233L0, 30233L1, 30233M0, 30233M1, 30233N1, 30233N1, 30233R0, 30233R1, 30233W0, 30233W1, 30240H0, 0240H1, 30240K0, 30240K1, 30240L0, 30240L1, 30240M0, 30240M1, 30240N0, 30240N1, 30240P0, 30240P1, 30240R0, 30240R1, 30240W0, 30240W1, 30243H0, 30243H1, 30243J0, 30243J1, 30243K0, 30243K1, 30243L0, 30243L1, 30243M0, 30243M1, 30243N0, 30243N1, 30243P0, 30243P1, 30243R0, 30243R1, 30243W0, 30243W1 |
| Mechanical Circulatory Support | ECMO: 39.65  Intra-aortic balloon pump: 37.61  Peripheral LVAD: 37.68 | ECMO: 5A15223, 5A1522F, 5A1522G, 5A1522H  Intra-aortic balloon pump: 5A02110, 5A02210  Peripheral LVAD: 5A0211D, 5A0221D, 02HA3RJ, 02HA4RJ |
|  |  |  |
|  |  |  |
| Outcomes |  |  |
| Procedural Hemorrhage | 998.11, 998.12, 285.1, 568.81 | I97.4*, I97.60 – I97.638, D62, L76.22, M96.81, M96.83, M96.811, K66.1 |
| Gastrointestinal Bleeding | 578.0, 562.03, 562.02, 537.84, 569.3, 569.13, 562.12, 569.85, 530.21, 531.00, 531.01, 531.20, 531.21, 531.40, 531.41, 531.60, 531.61, 532.00, 532.01, 532.20, 532.21, 532.40, 532.41, 532.60, 532.61, 533.00, 533.01, 533.20, 533.21, 533.40, 533.41, 533.60, 533.61, 534.00, 534.01, 534.20, 534.21, 534.40, 534.41, 534.60, 534.61, 535.01, 535.11, 535.21, 535.31, 535.41, 535.61, 535.71, 575.1, 578.9 | K92.0-92.2; K25.0-25.2; K25.4-25.6; K26.0-26.2; K27.0-27.2; K27.4-27.6; K28.0-28.2; K28.4-28.6, K57.01, K57.11, K57.21, K57.31, K57.41, K57.91, K55.21, K29.31, K29.21, K29.01, K29.41, K29.51, K29.61, K29.71, K29.81, K29.91, K31.82, K62.5, K20.81, K20.91 |
| Intracranial Bleeding | 430 431 432.xx | I60-62 |
| Stroke or Arterial Thromboemoblism | 433, 434, 435, 444, 445 | I163, I64, I65, I66, I174, I75 |
| Venous Thromboembolism | 453, 415 | I26, I82 |
| Sepsis or Catheter-Related Infection | 03.8*, 995.91, 995.92, 996.61, 996.62, 999.2*, 999.3*, | A40.*, A41.*, R65.2*, T802*, T826* |

**Supplemental Table 2: Cancer Types and Definitions**

|  | ICD-9 Code | ICD-10 Code |
| --- | --- | --- |
| Solid Cancer |  |  |
| Colorectal | 153, 154 | C18, C19, C20 |
| Upper GI Cancer | 150, 151, 152 | C15, C16, C17 |
| Non-Luminal GI Cancer | 155, 156, 157 | C22, C23, C24, C25, C26 |
| HEENT | 140 – 149, 160, 161 | C30, C31, C32, C33 |
| Lung | 162 | C34 |
| Mediastinal, Pleural, Cardiac Primary | 163, 164, 165 | C37, C38, C39 |
| Melanoma | 172 | C43 |
| Breast | 174, 175 | C50 |
| Gynecologic | 179 - 184 | C53, C54, C56 |
| Male Genitourinary (Penile, scrotal, testicular) | 187, 186 | C60, C62, C63 |
| Prostate | 185 | C61 |
| Genitourinary | 188, 189 | C64, C67, C66, C65, C68 |
| CNS Cancer | 190, 191, 192 | C69, C70, C71, C72 |
| Endocrine Tumors | 193, 194 | C73, C74, C75 |
| Soft Tissue/Sarcomas | 170, 171, 176 | C40, C41, C45, C46, C47, C48, C49 |
|  |  |  |
| *Hematologic Cancer* |  |  |
| Hodgkin Lymphoma | 201 | C81 |
| Non-Hodgkin Lymphoma | 200, 202 | C82, C83, C84, C85, C86, C88 |
| Plasma Cell Neoplasms (MM, Amyloid) | 203 | C90 |
| MPNs/MDS | 207.10-207.12, 205.10-205.12, 206.10 – 206.12, 238.71, 238.76 | C9210, C473, D7581, D45, D47.1, D47.3-D47.4 |
| Lymphoid Leukemia | 204 | C91 |
| Acute Myeloid Leukemia | 205, 206, 207, 208 | C92, C93, C94, C95 |
| Brain Metastasis or Primary Tumor | 191, 198.3 | C79.3, C71 |

**Supplemental Table 3: Patient Characteristics Prior to Propensity Score Matching**

|  | No Cancer  N = 40,730 | Cancer  N = 1,304 | SMD |
| --- | --- | --- | --- |
| Age, mean (SD) | 65.7 (12.2) | 69.5 (11.3) | 0.324 |
| Female sex, N (%) | 12,671 (31.1) | 368 (28.2) | 0.063 |
| Non-White Race, N (%) | 13,987 (34.3) | 368 (28.2) | 0.132 |
|  |  |  |  |
| Co-Morbidities |  |  |  |
| Prior MI | 2,808 (6.9) | 108 (8.3) | 0.052 |
| Prior PCI | 2,940 (7.2) | 102 (7.8) | 0.023 |
| Prior CABG | 1,036 (2.5) | 25 (1.9) | 0.042 |
| Heart Failure | 1,801 (4.4) | 58 (4.4) | 0.001 |
| Atrial Fibrillation | 9,533 (23.4) | 350 (26.8) | 0.079 |
| Anemia | 7,662 (18.8) | 350 (26.8) | 0.192 |
| Chronic Lung Disease | 7,757 (19.0) | 311 (23.8) | 0.117 |
| Smoking | 11,276 (27.7) | 329 (25.2) | 0.056 |
| Diabetes | 14,540 (35.7) | 411 (31.5) | 0.089 |
| Obesity | 5,531 (13.6) | 122 (9.4) | 0.133 |
| Hypertension | 20,452 (50.2) | 656 (50.3) | 0.002 |
| Liver Disease | 679 (1.7) | 35 (2.7) | 0.070 |
| Peripheral Vascular Disease | 4,640 (11.4) | 147 (11.3) | 0.004 |
| CKD | 7,685 (18.9) | 273 (20.9) | 0.052 |
| Prior Stroke | 1,137 (2.8) | 32 (2.5) | 0.021 |
| Prior VTE | 439 (1.1) | 37 (2.8) | 0.127 |
| Thrombocytopenia | 5,188 (12.7) | 186 (14.3) | 0.045 |
|  |  |  |  |
| Hospitalization Characteristics, N (%) |  |  |  |
| STEMI Presentation | 27,932 (68.6) | 864 (66.3) | 0.050 |
| Cardiac Arrest | 7,722 (19.0) | 201 (15.4) | 0.094 |
| Mechanical Complication | 1,095 (2.7) | 19 (1.5) | 0.087 |
| MCS Type, N (%) |  |  |  |
| IABP | 36,790 (90.3) | 1,166 (89.4) | 0.030 |
| pLVAD | 4,520 (11.1) | 152 (11.7) | 0.018 |
| ECMO | 1,215 (3.0) | 25 (1.9) | 0.069 |
| PCI | 22,822 (56.0) | 769 (59.0) | 0.059 |
| CABG | 10,661(26.2) | 276 (21.2) | 0.118 |
| Invasive Hemodynamic Monitoring | 16,608 (40.8) | 604 (46.3) | 0.112 |
| Mechanical Ventilation | 19,170 (47.1) | 665 (51.0) | 0.079 |
| Vasopressor Used | 3,294 (8.1) | 121 (9.3) | 0.042 |
| Palliative Care | 2,407 (5.9) | 113 (8.7) | 0.106 |
| DNR Status | 2,668 (6.6) | 123 (9.4) | 0.106 |
| Medicare or Medicaid | 24,793 (60.9) | 952 (73.0) | 0.260 |

**Supplemental Table 4: Patient and Hospitalization Characteristics After Propensity Score Matching Stratified by Cancer Type**

|  | No Cancer  N = 12,870 | Solid Cancer  N = 778 | Heme Cancer  N = 490 | SMD  No Cancer vs Solid Cancer | SMD  No Cancer vs Heme Cancer |
| --- | --- | --- | --- | --- | --- |
| Age, mean (SD) | 69.4 (11.7) | 69.9 (10.3) | 68.5 (12.8) | 0.046 | 0.072 |
| Female sex, N (%) | 3,688 (28.7) | 220 (28.3) | 141 (28.8) | 0.008 | 0.003 |
| Non-White Race, N (%) | 3,652 (28.4) | 217 (27.9) | 141 (28.8) | 0.011 | 0.009 |
|  |  |  |  |  |  |
| Co-Morbidities |  |  |  |  |  |
| Prior MI | 1,019 (7.9) | 69 (8.9) | 35 (7.1) | 0.034 | 0.029 |
| Prior PCI | 1,008 (7.8) | 69 (8.9) | 29 (5.9) | 0.037 | 0.076 |
| Prior CABG | 268 (2.1) | 13 (1.7) | 9 (1.8) | 0.030 | 0.018 |
| Heart Failure | 569 (4.4) | 29 (3.7) | 26 (5.3) | 0.035 | 0.041 |
| Atrial Fibrillation | 3,399 (26.4) | 200 (25.7) | 137 (28.0) | 0.016 | 0.035 |
| Anemia | 3,362 (26.1) | 202 (26.0) | 133 (27.1) | 0.004 | 0.023 |
| Chronic Lung Disease | 3,008 (23.4) | 205 (26.3) | 98 (20.0) | 0.069 | 0.082 |
| Smoking | 3,234 (25.1) | 204 (26.2) | 121 (24.7) | 0.025 | 0.010 |
| Diabetes | 4,039 (31.4) | 226 (29.0) | 171 (34.9) | 0.051 | 0.075 |
| Obesity | 1,171 (9.1) | 69 (8.9) | 50 (10.2) | 0.008 | 0.037 |
| Hypertension | 6,501 (50.5) | 403 (51.8) | 231 (47.1) | 0.026 | 0.067 |
| Liver Disease | 315 (2.4) | 21 (2.7) | 11 (2.2) | 0.016 | 0.013 |
| Peripheral Vascular Disease | 1,502 (11.7) | 87 (11.2) | 56 (11.4) | 0.015 | 0.008 |
| CKD | 2,683 (20.8) | 150 (19.3) | 115 (23.5) | 0.039 | 0.063 |
| Prior Stroke | 336 (2.6) | 18 (2.3) | 12 (2.4) | 0.019 | 0.010 |
| Prior VTE | 246 (1.9) | 18 (2.3) | 10 (2.0) | 0.028 | 0.009 |
| Thrombocytopenia | 1,795 (13.9) | 99 (12.7) | 81 (16.5) | 0.036 | 0.072 |
|  |  |  |  |  |  |
| Hospitalization Characteristics, N (%) |  |  |  |  |  |
| STEMI Presentation | 8,520 (66.2) | 535 (68.8) | 306 (62.4) | 0.055 | 0.078 |
| Cardiac Arrest | 2,018 (15.7) | 126 (16.2) | 73 (14.9) | 0.014 | 0.022 |
| Mechanical Complication | 180 (1.4) | 12 (1.5) | 7 (1.4) | 0.012 | 0.003 |
| MCS Type, N (%) |  |  |  |  |  |
| IABP | 11,522 (89.5) | 699 (89.8) | 438 (89.4) | 0.011 | 0.004 |
| pLVAD | 1,492 (11.6) | 90 (11.6) | 55 (11.2) | 0.001 | 0.012 |
| ECMO | 247 (1.9) | 10 (1.3) | 14 (2.9) | 0.050 | 0.061 |
| PCI | 7,557 (58.7) | 450 (57.8) | 292 (59.6) | 0.018 | 0.018 |
| CABG | 2,703 (21.0) | 145 (18.6) | 128 (26.1) | 0.059 | 0.121 |
| Invasive Hemodynamic Monitoring | 5,688 (44.2) | 329 (42.3) | 250 (51.0) | 0.039 | 0.137 |
| Mechanical Ventilation | 6,567 (51.0) | 387 (49.7) | 258 (52.7) | 0.026 | 0.033 |
| Vasopressor Used | 1,147 (8.9) | 69 (8.9) | 48 (9.8) | 0.002 | 0.030 |
| Palliative Care | 1,044 (8.1) | 75 (9.6) | 30 (6.1) | 0.054 | 0.077 |
| DNR Status | 1,128 (8.8) | 67 (8.6) | 46 (9.4) | 0.005 | 0.022 |
| Medicare or Medicaid | 9,392 (73.0) | 586 (75.3) | 334 (68.2) | 0.054 | 0.106 |

**Supplemental Table 5: Outcomes of Patients with versus without Cancer After PSM and Stratification by Cancer Type**

|  | No Cancer  N = 12,870 | Cancer  N = 1,287 | Solid Cancer  N = 778 | Heme Cancer  N = 490 | P value  vs Any Cancer | P Value  Vs Solid Cancer | P Value  Vs Heme Cancer |
| --- | --- | --- | --- | --- | --- | --- | --- |
| Primary Outcome |  |  |  |  |  |  |  |
| Death | 4,199 (32.6) | 419 (32.6) | 269 (34.6) | 144 (29.4) | 0.97 | 0.27 | 0.14 |
|  |  |  |  |  |  |  |  |
| Secondary Outcomes |  |  |  |  |  |  |  |
| Thrombotic Complications ^a^ | 1,147 (8.9) | 125 (9.7) | 77 (9.9) | 48 (9.8) | 0.33 | 0.36 | 0.52 |
| Major Bleeding ^b^ | 4,090 (31.8) | 484 (37.6) | 282 (36.2) | 195 (39.8) | < 0.001 | 0.010 | < 0.001 |
| Sepsis or Catheter-Related Infection | 1,581 (12.3) | 158 (12.3) | 94 (12.1) | 63 (12.9) | 1.00 | 0.91 | 0.67 |
| Procedural Bleeding | 2,151 (16.7) | 267 (20.7) | 152 (19.5) | 111 (22.6) | < 0.001 | 0.043 | 0.001 |
| Intracranial Bleeding | 78 (0.6) | 9 (0.7) | 3 (0.4) | 6 (1.2) | 0.71 | 0.63 | 0.13 |
| Gastrointestinal Bleeding | 743 (5.8) | 86 (6.7) | 53 (6.8) | 31 (6.3) | 0.19 | 0.24 | 0.62 |
| Transfusion | 2,213 (17.2) | 275 (21.4) | 161 (20.7) | 111 (22.6) | < 0.001 | 0.015 | 0.002 |
| Stroke or Arterial Thromboembolism | 763 (5.9) | 74 (5.7) | 40 (5.1) | 34 (6.9) | 0.85 | 0.43 | 0.33 |
| Venous Thromboembolism | 437 (3.4) | 60 (4.7) | 42 (5.4) | 18 (3.7) | 0.021 | 0.005 | 0.70 |

^a^ Composite of stroke or arterial thromboembolism or VTE

^b^ Composite of procedural bleeding, intracranial bleeding, gastrointestinal bleeding, or transfusion of blood products

**Supplemental Table 6: Logistic Regression of Outcomes Stratified by Cancer Type after PSM**

|  | Solid Cancer  Odds Ratio (95% Confidence Interval) | Hematology Cancer  Odds Ratio (95% Confidence Interval) | Hematology Cancer  Adjusted Odds Ratio (95% Confidence Interval) ^a^ |
| --- | --- | --- | --- |
| Primary Outcome |  |  |  |
| Death | 1.09 (0.94 – 1.27) | 0.86 (0.71 – 1.05) | 0.91 (0.75 – 1.12) |
|  |  |  |  |
| Secondary Outcomes |  |  |  |
| Thrombotic Complications | 1.12 (0.88 – 1.43) | 1.11 (0.82 – 1.50) | 1.07 (0.79 – 1.45) |
| Major Bleeding | 1.22 (1.05 – 1.42) | 1.42 (1.18 – 1.71) | 1.35 (1.11 – 1.64) |
| Sepsis or Catheter-Related Infection | 0.98 (0.79 – 1.23) | 1.05 (0.80 – 1.38) | 1.04 (0.79 – 1.36) |
| Procedural Bleeding | 1.21 (1.01 – 1.45) | 1.46 (1.18 – 1.81) | 1.34 (1.06 – 1.69) |
| Intracranial Bleeding | 0.63 (0.20 – 2.02) | 2.03 (0.88 – 4.69) | 2.16 (0.93 – 4.98) |
| Gastrointestinal Bleeding | 1.19 (0.89 – 1.59) | 1.10 (0.76 – 1.60) | 1.14 (0.79 – 1.66) |
| Transfusion | 1.26 (1.05 – 1.50) | 1.41 (1.14 – 1.75) | 1.35 (1.08 – 1.69) |
| Stroke or Arterial Thromboembolism | 0.86 (0.62 – 1.19) | 1.18 (0.83 – 1.69) | 1.15 (0.80 – 1.64) |
| Venous Thromboembolism | 1.62 (1.17 – 2.35) | 1.08 (0.67 – 1.75) | 1.03 (0.63 – 1.66) |

^a^ Adjusted for CABG, invasive hemodynamic monitoring and insurance status
